# Non-Communicable Diseases and Associated Risk Factors in Burning Mouth Syndrome Patients

**DOI:** 10.3390/medicina59122085

**Published:** 2023-11-27

**Authors:** Ioanina Parlatescu, Cosmin Dugan, Bogdan Ovidiu Popescu, Serban Tovaru, Maria Dobre, Elena Milanesi

**Affiliations:** 1 Faculty of Dentistry, Carol Davila University of Medicine and Pharmacy, 050474 Bucharest, Romania; ioanina.parlatescu@umfcd.ro (I.P.); serban.tovaru@gmail.com (S.T.); 2 Faculty of Medicine, Carol Davila University of Medicine and Pharmacy, 050474 Bucharest, Romania; cosmin.dugan@drd.umfcd.ro (C.D.); bogdan.popescu@umfdc.ro (B.O.P.); elena.milanesi@ivb.ro (E.M.); 3 Victor Babeș National Institute of Pathology, 050096 Bucharest, Romania

**Keywords:** burning mouth syndrome, noncommunicable diseases, cardiovascular diseases, hypertension, dyslipidemia

## Abstract

*Background and Objectives*: Noncommunicable diseases (NCDs) are a group of non-transmissible conditions that tend to be of long duration and are the result of a combination of genetic, physiological, environmental, and behavioral factors. Although an association between oral disorders and NCDs has been suggested, the relationship between Burning Mouth Syndrome (BMS) and NCDs and their associated risk factors has not been deeply investigated. In this study, we aim to identify associations between BMS and NCDs in the Romanian population. *Materials and Methods*: Ninety-nine BMS patients and 88 age-matched controls (aged 50 and over) were clinically evaluated for the presence of eight noncommunicable diseases (NCDs) and their most common risk factors, including hypertension, dyslipidemia, smoking, and obesity. *Results*: The results of our study showed that the BMS in the Romanian population seems to be significantly associated with cardiovascular diseases (CVDs) (*p* < 0.001) and two of their risk factors, hypertension (*p* < 0.001) and dyslipidemia (*p* < 0.001). Moreover, evaluating the Framingham Risk Score (FRS) in the individuals not affected by CVDs (73 CTRL and 38 BMS), we found that 13.2% of BMS patients reported a moderate risk of developing CVDs in ten years, compared to the controls, all of whom presented a low risk (*p* = 0.002). *Conclusions*: Our findings suggest that a multidisciplinary clinical approach, which also includes a cardiovascular evaluation, is essential for the successful management of BMS. Moreover, these data highlighted the importance of introducing an integrated strategy for the prevention and care of NCDs in BMS patients.

## 1. Introduction

Non-communicable diseases (NCDs) are chronic diseases that are not transmitted from person to person. They are the results of a combination of different factors, including genetic, physiological, environmental, and behavioral factors, with the last two being modifiable through a comprehensive approach to prevention and control. The reduction of premature mortality from NCDs by one-third through prevention and treatment is one of the challenges facing WHO by 2030 [1]. The term NCDs covers a large range of health problems, with cardiovascular diseases (CVDs) being the leading contributors to the global burden of disease among the NCDs, followed by cancer, chronic respiratory diseases, and diabetes mellitus [2]. One important matter is that NCDs do not simply heal, and a full recovery is uncommon, thus being key contributors to illness and disability in low- and middle-income countries [3]. 

Recent research has suggested a possible bidirectional relationship between oral diseases and systemic NCDs, identifying a strong association between periodontal disease and high risk for cancer, CVDs, diabetes, adverse pregnancy outcomes, lower longevity, and neurodegeneration. Moreover, the results of this meta-analysis have shown that dental, periodontal, and endodontic treatments, as well as mandibular advancement, positively impact different NCDs [4]. The investigation of the presence of chronic diseases in oral leukoplakia has shown an association with dyslipidemia, musculoskeletal diseases, and asthma [5]. 

Burning mouth syndrome (BMS) is a chronic oral disease characterized by pain affecting the oral mucosa without any clinical or biological disturbances [6,7]. The different terms used for this disorder include, but are not limited to, the following: burning mouth disease, “complex oral sensitivity disorder”, glossodinia, stomatodynia, glossopyrosis, stomatopyrosis, “hot tongue syndrome”, and “scalded mouth syndrome” [8]. The BMS estimated prevalence in the population-based studies was 1.73%, and in the clinical-based studies it was 7.72% [9]. It carries a strong female predilection [10]. The oral symptoms reported by BMS patients have a large range of variation, from burning, itching, pain, dry mouth, sialorrhea, and taste alterations to the unusual sensation of a foreign body on different mucosal areas [11]. Many BMS patients report that these symptoms decrease or disappear when eating and drinking [11,12]. Based on the evolution of the symptoms during the day, BMS is divided into three main clinical types: type I to type III. BMS type I describes the symptoms that are missing in the morning, appear and increase during the day, and are present in 35% of the patients. BMS type II includes patients who report symptoms present all day continuously and is associated with psychiatric disorders, especially chronic anxiety, and is encountered in 55% of BMS individuals. BMS type III is reported in 10% of patients and has an intermittent evolution with asymptomatic days and the usual location of the symptoms (throat, floor of the mouth, buccal mucosa) [13]. A multifactorial etiology is unanimously accepted and comprises central or peripheral neuropathy as well as a psychogenic component (psychological disorders or psychiatric diseases) [10].

BMS influences the well-being of patients and negatively impacts their oral health-related quality of life in connection with their emotional and social states [14]. The association between depression and anxiety was reported in these patients [15].

The NCDs and the risk factors associated with BMS have not been deeply investigated, with only a few studies conducted in different populations [16,17,18]. A case-control study on a Spanish cohort reported that in general, BMS patients suffer more comorbidities than controls, mainly represented by mental, behavioral, and neurodevelopmental disorders [16]. A larger study conducted in Italy, involving over 200 patients and 200 controls, has found a statistically significant higher proportion of BMS patients suffering from hypertension compared to controls [17]. The same group of authors analyzing a cohort of BMS women compared to not-affected women found that, in addition to hypertension, hypercholesterolemia was more frequent in the BMS women [18]. Another report on the Japanese population, investigating the prevalence of 13 diseases in BMS patients, found that most of the investigated diseases seemed to have little influence on the majority of patients with BMS [19]. 

Previous research has demonstrated a relationship between BMS and the central and/or peripheral neuropathic and psychogenic contributors [20,21]. One investigation that may shed some light on the cerebral etiology of BMS is functional brain imaging [19]. Notably, two research groups reported a correlation between brain abnormalities identified by MRI and the existence of BMS [22,23]. 

There is a knowledge gap, and research is needed to better understand the connection between BMS and these chronic, multifactorial diseases.

In this article, we report the results of a case-control study conducted on the Romanian population regarding the putative association between noncommunicable diseases, their main risk factors, and BMS.

## 2. Materials and Methods

### 2.1. Study Population

The individuals participating in this study were recruited at the Oral Medicine Department, Faculty of Dentistry, “Carol Davila” University of Medicine and Pharmacy in Bucharest. The target population consisted of BMS Romanian patients and controls (CTRL) aged 50 and over. The diagnosis of BMS was established in accordance with the guidelines of the WHO (World Health Organization, 2023) [6] and the International Headache Society (Headache Classification Committee of the International Headache Society (IHS), 2018) [7]. The inclusion criteria were: (i) individuals aged at least 50 years; (ii) the daily presence of at least one of the oral symptoms (burning sensation, pain, and itching sensation) for more than 2 h for at least 3 months; (iii) a normal clinical aspect of the oral mucosa and a negative oral fungal test; and (iv) biological tests (complete blood count, serum iron and ferritin, blood sugar levels, B1, B6, and B12 vitamins, as well as the thyroid panel) within a normal range. The CTRL group inclusion criteria were: (i) individuals aged at least 50; and (ii) the absence of BMS symptoms and oral mucosal lesions. Part of the described inclusion criteria was used in a previous study [24]. All the patients and controls with the following features were excluded from this study: (1) presence of oral mucosal diseases; (2) positivity at an oral fungal test and remission of the symptoms after treatment; and (3) dental, occlusal, and temporomandibular joint disorders inducing pain mimicking BMS symptomatology. As some authors have reported an association between BMS and the use of angiotensin-converting enzyme inhibitors (ACE inhibitors) [25,26], we referred the patients using ACE inhibitors to the cardiologists to replace this therapy. In 3 patients, the BMS symptoms remitted after 1 month of the replacement medication, and the patients were excluded from our study. 

We enrolled 99 BMS patients and 88 CTRL who were clinically evaluated for the presence of non-communicable diseases (NCDs) comprising hypertension (HT), cardiovascular diseases (CVDs), dyslipidemia (DL) (including hypertriglyceridemia and/or hypercholesterolemia), Type 1 and Type 2 diabetes mellitus (T1DM and T2DM), hyperthyroidism (HyperThy), hypothyroidism (HypoThy), autoimmune diseases (ADs), gastritis (Gas), and previous cancer with no evidence of disease at the moment of the enrollment in this study (Prev_Canc). Patients were considered to have HT when their systolic blood pressure was ≥140 mm Hg and/or their diastolic blood pressure (DBP) was ≥90 mm Hg following repeated examinations. The hypertension grading was also considered: Grade 1 (140–159 and/or 90–99 mmHg), Grade 2 (160–179 and/or 100–109 mmHg), and Grade 3 (>180 and/or >110 mmHg). 

The CVDs encountered were ischemic cardiomyopathy, carotid adenomatosis, and the presence of coronary stents. 

At the moment of the recruitment, all patients and controls were under treatment to control the symptoms of the reported NCDs. 

Moreover, for 73 controls and 38 BMS patients, all without CVDs, the Framingham risk score (FRS) to predict the 10-year cardiovascular disease risk (%) has been calculated. Risk was considered low if the FRS was <10%, moderate if the FRS = 10–19%, and high if the FRS was ≥20%. Information on sociodemographic characteristics was also recorded and comprised: sex, age, BMI, level of education, marital status, and employment status. 

Written informed consent was obtained from all the participants, and this study protocol was approved by the scientific research ethics committee of Carol Davila” University (approval number 36988/2022), ensuring that it conformed to the ethical guidelines of the 1975 Declaration of Helsinki.

### 2.2. Statistical Analysis 

All the analyzed features are presented with descriptive statistics like means ± standard deviation (SD) or number (percentage). Two sample *t*-test student were used to compare continuous variables, and the chi-square test and the Fisher’s exact test were used for categorical variables. The level of statistical significance was set at *p* < 0.05. Analyses were performed using IBM SPSS v25 (Chicago, IL, USA).

## 3. Results

The mean age of participants was 64.03 years (SD = 7.833; min–max = 50–85), and most were female (82.9%). No difference in sex distribution, mean age, BMI, marital status, or employment status (*p* > 0.05) was observed between BMS patients and controls, and the groups were not homogeneous for the level of education (*p* < 0.001) (Table 1).

The main form of BMS in our study group was type II (55.6%), followed by type I (34.3%), and type III (10.1%). The main symptom reported by most of the patients was the burning sensation, encountered in 63 cases. Moreover, most of the patients reported an intermittent frequency of symptoms (52.5%). Among the oral sites involved, the tongue mucosa was the most frequent (77.7%). Regarding food sensitivity, most of the patients (33.3%) reported sensitivity to sour and acidic foods. The characteristics of the BMS patients are reported in Table 2.

The presence of NCDs and risk factors among the BMS patients and controls is presented in Table 3. As reported in the table, we observed a significantly higher frequency of individuals with hypertension and dyslipidemia in the BMS group (84.8% and 85.6%, respectively) compared to the controls (*p* < 0.001). When considering the comparison of the frequency of NCDs between patients and controls, we found a higher frequency of cardiovascular diseases (*p* < 0.001), autoimmune diseases (*p* = 0.033), gastritis (*p* = 0.003), and history of previous cancer (*p* = 0.002) with no evidence of disease at the moment of enrollment in this study.

Most of the BMS patients with HT reported grade 2 hypertension (n = 71, 84.5%), followed by grade 3 (n = 9, 10.7%), and grade 1 (n = 4, 4.8%). The 16 CTRLs with HT reported grade 2 hypertension (n = 8, 50.0%), followed by grade 3 (n = 6, 37.5%), and grade 1 (n = 2, 12.5%). Regarding the CVDs, 46 BMS patients (85.2%) and 14 CTRL patients (100%) reported ischemic cardiomyopathy. The rest of BMS patients reported the following CVDs: carotid adenomatosis (n = 3, 5.6%) and the presence of coronary stents (n = 5, 9.2%).

## 4. Discussion

In this study, we investigated the presence of NCDs and their risk factors in a large cohort of BMS patients and age-matched controls (aged 50 and over) in order to identify associations between BMS and NCDs in the Romanian population. BMS is a challenging disease for both the patients and the multidisciplinary medical team, which tries to provide the best quality patient care. The BMS multifactorial etiology raised the need for large and detailed investigations, which include oral mucosa and dental evaluation, biochemical serological tests, and cerebral MRI to identify putative white matter hyperintensities. 

Despite the effort of the last decade in identifying the main factors leading to the BMS and influencing its evolution, the results are still contrasting and non-homogenous [27,28]. Chimenos-Kustner et al. reported a statistically significant association of BMS with triggering factors, parafunctional habits, and oral hygiene [28].

In this context, the association between BMS and a large panel of NCDs, together with their risk factors, has not been deeply investigated. To our knowledge, the available literature on this topic refers to studies involving a relatively small number of individuals, investigating a limited number of NCDs and risk factors.

Our results obtained from a cohort of 187 individuals (99 BMS patients and 88 controls) show that the BMS in the Romanian population is associated with cardiovascular diseases and their risk factors, hypertension, and dyslipidemia. 

Data from Romania’s National Institute for Public Health indicate that hypertension, smoking, high cholesterol, obesity, alcohol use, a low diet of fruits and vegetables, and physical inactivity are the main risk factors for cardiovascular diseases (CVDs) [29]. Moreover, heart disorders account for 60% of Romanian mortality, and hypertension is a risk factor present in 45.1% of the Romanian general adult population [29]. 

In our study, the presence of HT in the BMS group was 84.8%, which is consistently higher than the frequency identified in studies conducted in other countries. For example, Adamo and collaborators reported a HT frequency of 55% in BMS Italian patients [17], with a lower frequency of 51.2% considering only the female patients [18]. In line with our results, these authors also found a significantly higher frequency of HT in BMS patients compared to controls [17,18]. A consistent lower frequency of HT was observed in the BMS Japanese population, with 19% of patients reporting HT [19]. 

The second risk factor for CVDs identified in our study associated with BMS was dyslipidemia. This lipidic disorder is a contributing factor in atherosclerosis and cardiovascular diseases [30], and it has been estimated that 67.1% of Romanian adults have at least one lipid abnormality [31]. Our findings revealed dyslipidemia in 85.6% of BMS patients versus 39.8% of controls. In line with our results, Jin, Jian-Qiu, et al. [32], analyzing Chinese BMS patients, found a significant association between BMS and the levels of total cholesterol and triglycerides. These results have been partially reproduced in Italian BMS women, who reported a higher percentage of hypercholesterolemia compared to healthy women [18].

CVDs, which have been registered in 44.5% of BMS and 15.9% of controls, are the only NCDs we found associated with BMS in the Romanian population (*p* < 0.001). According to our knowledge, this association is reported for the first time in our study. On the contrary, Adamo and collaborators have not found a significant association between BMS and myocardial infarction and other CVDs in the Italian BMS patients [17], and only 9.2% of BMS Japanese patients reported heart disease [19]. 

In addition, considering the patients without CVDs, we also calculated the Framingham risk score (FRS), which serves as a predictor of cardiovascular disease risk over the next ten years for asymptomatic individuals. Age, gender, blood pressure, total cholesterol level, high-density lipoprotein level, diabetes, and smoking are the variables included in this risk prediction model [33]. Considering that BMS is more frequent in females than males, this test has been chosen due to its superior discrimination for women over men [33]. A statistically significant association between FRS and BMS was found, with 13.2% of patients reporting a moderate risk of developing CVD in ten years, compared to the controls, all of whom presented a low risk (*p* = 0.002). 

Since there is a relationship between increased blood sugar levels and the reduced ability of nerve fibers in the autonomic, peripheral, and central nervous systems to operate [34], the presence of diabetes in association with BMS has also been evaluated in this study. No significant association between the presence of diabetes and BMS has been found in our cohort. This negative result is in line with the findings of Sardella et al., who, in a prospective case-control study, did not find any association between BMS and diabetes [27]. On the contrary, an American study reported increased blood glucose levels in 23.7% of BMS cases compared to the general population, where the prevalence of diabetic mellitus is 9.3% [35].

As the thyroid hormones have an important role in the metabolism, nervous system, and taste papillae growth, a thyroid abnormality evaluation is a key point included in the first evaluation of a BMS patient. Morr Verenzuela et al. found that 5.2% of BMS patients presented increased levels of TSH, while 3.2% had low abnormal TSH values [35]. This data have been confirmed by a systematic review, assessing that thyroid hormone alteration levels represent a factor to be considered in the secondary BMS [36]. In contrast with the reported findings, our study did not find any association with thyroid dysfunction, with only one patient presenting hyperthyroidism. A comparative analysis with previous research on the risk factors and general comorbidties included in NCDs is presented in Table 4.

## 5. Conclusions

Our study, showing that the BMS in the Romanian population is significantly associated with cardiovascular diseases, hypertension, and dyslipidemia, points out that a multidisciplinary clinical approach, which also includes a cardiovascular evaluation, is essential for the successful management of BMS. Moreover, our results highlighted the importance of an integrated strategy for the prevention and care of NCDs in the BMS population. Further studies to better understand the crosstalk between NCDs and BMS are needed.

## Figures and Tables

**Table 1:** Sociodemographic data of the participants.

	CTRL (N = 88)	BMS (N = 99)	Significance
Age in years (mean ± SD)	63.44 ± 7.53	64.55 ± 8.09	^#^ *p* = 0.338
Sex (F%; M%)	81.8% F; 18.2% M	83.8% F; 16.2% M	^$^ *p* = 0.714
Education in years (mean ± SD)	13.78 ± 1.06	14.76 ± 1.72	^#^ *p* < 0.001
Marital status (%)	Single = 5.7%	Single = 3.0%	^$^ *p* = 0.773
	Married = 84.1%	Married = 83.8%	
	Widow/er = 6.8%	Widow/er = 9.1%	
	Divorced = 3.4%	Divorced = 4.0%	
Employment status (%)	Unemployed = 1.1%	Unemployed = 1.0%	^$^ *p* = 0.987
	Retired = 68.2%	Retired = 70.7%	
	Part-time job = 5.7%	Part-time job = 6.1%	
	Full-time job = 19.3%	Full-time job = 16.2%	
	Own business = 5.7%	Own business = 6.1%	

^#^ *t*-student test; ^$^ chi-square test.

**Table 2:** General features of the BMS cohort.

BMS classification (Type I, II, III)	Type I = 34 (34.3%)
	Type II = 55 (55.6%)
	Type III = 10 (10.1%)
Main symptom	Burning sensation = 63 (63.6%)
	Pain = 31 (31.3%)
	Itching sensation = 5 (5.1%)
Symptoms frequency	Rare = 8 (8.1%)
	Intermittent = 52 (52.5%)
	Frequent = 33 (33.3%)
	Constant = 6 (6.1%)
Oral site involved	Tongue = 77 (77.7%)
	Tongue + gums = 10 (10.1%)
	Tongue + pharynx = 7 (7.1%)
	Other = 5 (5.1%)
Food sensitivity	Sour/acid sensitivity = 33 (33.3%)
	Sour and sweet sensitivity = 26 (26.3%)
	Hot/spicy sensitivity = 12 (12.1%)
	Sweet sensitivity = 11 (11.1%)
	Tooth brushing sensitivity = 11 (11.1%)
	Generalized sensitivity = 3 (3.05%)
	Without alteration, 3 (3.05%)
Taste affection	No = 65 (65.7%)
	Yes = 34 (34.3%)
VAS-Visual analogue scale (0–10)	Mild pain (1–3) = 9 (9.1%)
	Moderate pain (4–6) = 90 (90.9%)

**Table 3:** Non-communicable diseases and risk factors in this study cohorts.

Variable	CTRL (N = 88) n (%)	BMS (N = 99) n (%)	Odds Ratio (95% CI)	*p*-Value (Chi-Square Test)
**Risk factors**				
HT	16 (18.2%)	84 (84.8%)	25.2 (11.6–54.5)	*p < 0.001*
Smokers	28 (31.8%)	31 (31.3%)	0.97 (0.53–1.81)	*p* = 0.941
BMI category			– –	*p* = 0.680
BMI 18.5–24.9 normal	23 (26.2%)	22 (22.3%)		
BMI 25.0–29.9 overweight	51 (57.9%)	57 (57.5%)		
BMI > 29 obesity	14 (15.9%)	20 (20.2%)		
DL	35 (39.8%)	85 (85.6%)	9.2 (4.5–18.7)	*p < 0.001*
**NCDs**				
CVD	14 (15.9%)	54 (44.5%)	6.3 (3.2–12.7)	*p < 0.001*
T1DM	4 (4.5%)	3 (3.0%)	0.6 (0.1–3.0)	*p* = 0.708
T2DM	3 (3.4%)	11 (11.1%)	3.5 (0.9–13.1)	*p* = 0.054
HypoThy	2 (2.3%)	0	0.5 (0.4–0.5)	*p* = 0.220
HyperThy	0	1 (1%)	0.5 (0.4–0.6)	*p* = 1
Ads	3 (3.4%)	12 (12.1%)	3.9 (1.1–14.3)	*p = 0.033*
Gas	1 (1.1%)	12 (12.1%)	12.0 (1.5–94.3)	*p = 0.003*
Prev_Canc	1 (1.1%)	13 (13.1%)	13.1 (1.68–102.7)	*p = 0.002*
Framingham Risk Score (FRS) *			– –	*p = 0.002*
FRS < 10% (Low Risk)	100%	86.8%		
FRS 10–19% (Medium Risk)	0%	13.2%		
FRS ≥ 20% (High Risk)	0%	0%		

* available for 73 CTRL and 38 BMS asymptomatic for CVD.

**Table 4:** Comparation of our results with previous research.

	Study Type	Hypertension	Cardiovascular Diseases	Dyslipidemia	Diabetes	Thyroid Disorders
Our results 88 BMS patients vs. 99 controls	Case-control	Significant differences 84.8% BMS patients vs. 18.2% controls	Significant differences 44.5% BMS vs. 15.9% controls	Significant differences 85.6% BMS vs. 39.8% controls	No significant differences. 14.1% BMS vs. 7.9% controls	No significant differences. Hypothyroidism 2.3% BMS vs. 0 controls
Adamo et al., 2023 [17] 242 BMS patients vs. 242 controls	Case control	Significant differences (55% BMS vs. 33.5%controls)	No significant differences. Other cardiovascular diseases 9.5% BMS vs. 7.4% controls Myocardial infarction 6.6% BMS vs. 3.3% controls	No significant differences. Hypercolesterolemia 34.3% BMS vs. 28.5% controls	*	No significant differences. Hypothyroidism 12% BMS vs. 9.1% controls
Canfora et al., 2023 [18] 250 BMS patients women vs. 250 healthy women	Case-control	Statistically significant differences (51.2% BMS vs. 30.4% controls)	No significant differences. Other cardiovascular diseases 6% BMS vs. 5.6% controls Myocardial infarction 1.6% BMS vs. 2.4% controls	Statistically significant differences (36% BMS vs. 21.2% controls)	*	Hypothyroidism18.4% BMS vs. 13.6% controls Hyperthyroidism 2% BMS vs. 1.6% controls
Suga, 2019 [19] 1543 BMS patients	Cross-sectional	19.0% in BMS patients	9.2% in BMS patients	Hyperlipidemia in 17.2% BMS patients	In 4.5% BMS patients	In 6.4% BMS patients
Jin, Jian-Qiu et al., 2020 [32] 352 BMS patients 391 controls	Case-control	No significant differences. 27.8 BMS vs. 23.5% controls	*	Statistically significant differences for total cholesterol values	No significant differences.	*
Morr Verenzuela et al., 2017 [35] 659 BMS patients	Cross-sectional	*	*	*	23.7% of patients -increased blood glucose	5.2% of patients-high TSH, 3.2%-low TSH
Sardella et al., 2006 [27] 61 BMS vs. 54 controls	Case-control	*	*	*	Not significant differences	*

* not applicable.

## Data Availability

The data presented in this study are available on reasonable request from the corresponding author.
